# Low Growth Hormone Levels Predict Poor Outcome of Hepatitis B Virus-Related Acute-on-Chronic Liver Failure

**DOI:** 10.3389/fmed.2021.655863

**Published:** 2021-07-06

**Authors:** Daxian Wu, Lingjian Zhang, Shanshan Ma, Yalei Zhao, Ronggao Chen, Fen Zhang, Qiuhong Liu, Xiaowei Xu, Zhongyang Xie

**Affiliations:** ^1^Department of Infectious Diseases, The First Affiliated Hospital of Nanchang University, Nanchang, China; ^2^Collaborative Innovation Centre for Diagnosis and Treatment of Infectious Diseases, College of Medicine, First Affiliated Hospital, Zhejiang University, Hangzhou, China; ^3^State Key Laboratory for Diagnosis and Treatment of Infectious Diseases, School of Medicine, First Affiliated Hospital, Zhejiang University, Hangzhou, China; ^4^Department of Hepatobiliary and Pancreatic Surgery, School of Medicine, First Affiliated Hospital, Zhejiang University, Hangzhou, China; ^5^Department of Infectious Diseases, School of Medicine, First Affiliated Hospital, Zhejiang University, Hangzhou, China

**Keywords:** growth hormone, hepatitis B, acute-on-chronic liver failure, mortality, prognostic model

## Abstract

**Background and Aims:** Hepatitis B virus-related acute-on-chronic liver failure (HBV-ACLF) remains a serious entity with high mortality. Growth hormone (GH) is related to the liver metabolism and regeneration. The present study aimed to explore the changes and prognostic efficacy of GH on the outcome of HBV-ACLF.

**Methods:** A prospective cohort of 124 patients and a cross-sectional cohort of 142 subjects were enrolled. GH and insulin-like growth factor-1(IGF-1) were detected by ELISA. Thirty-day survival was collected and the association between GH and the 30-day mortality of HBV-ACLF was analyzed.

**Results:** The mean age of the whole prospective cohort was 46.61 ± 12.71 years, and 19 (15.3%) patients were female. The median (IQR) of GH levels in non-survivors were 1106.55 (674.25, 1922.4) pg/ml, which were significantly lower than in survivors (*p* < 0.001). In the cross-sectional cohort, GH level was significantly higher in liver cirrhosis - acute decompensation (LC-AD) group than liver cirrhosis (LC) group (*p* < 0.001) while IGF-1 decreased significantly in LC, LC-AD, ACLF groups than health control (HC) and chronic Hepatitis B (CHB) groups (*p* < 0.001). The area under the receiver operating characteristic curve (AUROC) of GH for predicting 30-day mortality was 0.793. We built a new prognostic model, namely MELD-GH, which showed better predictive efficacy than Child-Pugh, MELD, CLIF-SOFA, and CLIF-C ACLF scores.

**Conclusions:** Low GH predicted the poor outcome of HBV-ACLF patients. GH and IGF-1 levels were differently distributed among HC, CHB, LC, LC-AD, and ACLF patients. MELD-GH had better predictive accuracy when compared to Child-Pugh, MELD, CLIF-SOFA, and CLIF-C ACLF scores.

## Introduction

Currently in China, there are around 35 million patients with hepatitis B virus (HBV) infection, and the social burden caused by chronic hepatitis B (CHB) is still heavy (1). Moreover, the reactivation of HBV is the leading cause of acute-on-chronic liver failure (ACLF) (2, 3). ACLF is characterized as acute deterioration of liver function, organ failure and high short-term mortality in the presence of preexisting chronic liver diseases (CLDs) (4, 5), which are associated with organ failures and high short-term mortality.

So far, the pathophysiological mechanism of ACLF hasn't been elucidated clearly. Disordered immune function and liver regeneration are considered two factors to play key roles on the prognosis of ACLF patients (2). After the injury of a precipitating event, massive and sub-massive hepatic necrosis occurred (6), which caused excessive immune responses and pulled the trigger of liver regeneration. Recent studies suggested that, there might be two distinct regeneration patterns in livers with massive hepatic necrosis in patients with acute liver failure (7). Unfortunately, our knowledge on the mechanism of the regeneration is quite limited and need further study.

Growth hormone (GH), as one of the most important endocrine hormones in human body, plays an important role in promoting growth and regulating carbohydrate, lipid, protein, and mineral metabolism. It also plays a vital role on liver regeneration (8–10). Some studies have reported that there might be a correlation between GH and liver disease (11, 12). For example, in alcoholic liver disease and non-alcoholic liver disease, GH may be involved in the development of diseases and affect lipid metabolism in hepatocytes (13, 14). Some reports have also reported a reduction in GH concentrations in patients with reciprocal cirrhosis, which was associated with prognosis (15). Insulin-like-growth factor-1 (IGF-1), which is synthesized mainly in the liver, mediates most of the biological functions of GH (16) and is reported to participated in the process of liver regeneration (17). However, whether GH and IGF-1 regulate the liver regeneration and affect the prognosis of ACLF patients remains unknown.

In this study, we built a prospective cohort of HBV-ACLF patients, and identified differences in serum levels of GH and IGF-1 between survival and non-survival group and then explored their variability in a cross-sectional cohort. Furthermore, we built a prognostic model containing GH and evaluated its efficacy of for predicting short-term outcomes of HBV-ACLF patients.

## Methods

### Patients

**Figure 1:**
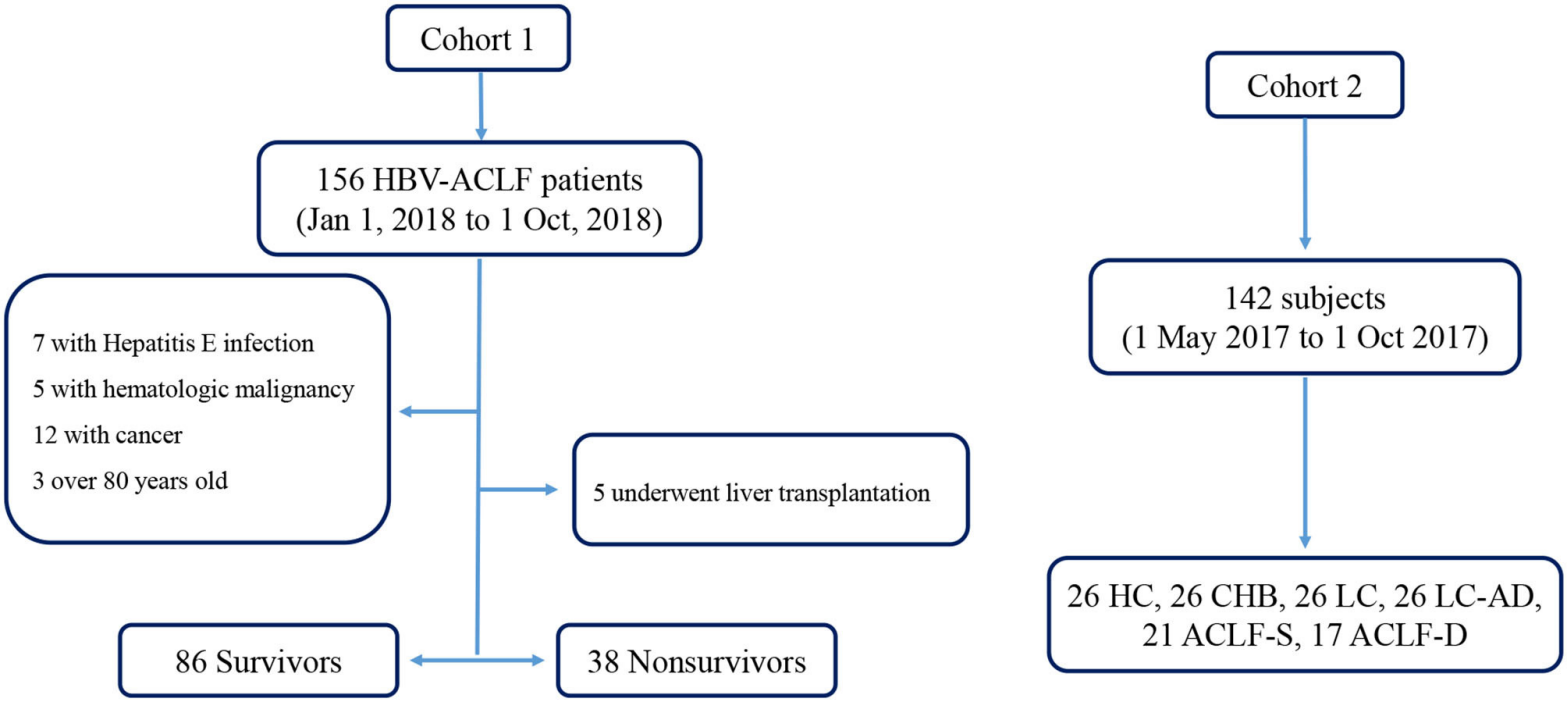
Flow chart of enrolled cohorts. ACLF-D, acute-on-chronic liver failure-death; ACLF-S, acute-on-chronic liver failure-survival; CHB, chronic Hepatitis B; HBV-ACLF, Hepatitis B related acute-on-chronic liver failure; HC, health control; LC, liver cirrhosis; LC-AD, liver cirrhosis-acute decompensation.

Two cohorts were enrolled in this study, and the flow chart was in Figure 1. In the first cohort, we enrolled HBV-ACLF patients who presented with a new episode of acute hepatic insult manifesting as acute jaundice, coagulopathy, ascites, upper gastrointestinal bleeding, and hepatic encephalopathy (HE) between 1 January 2018 and 1 October 2018. Patients were divided into survivor and non-survivor groups according to their survival status of 30 days after admission. Patients undergoing liver transplantation were excluded. In the second cohort, a cross-sectional investigation was performed using a cohort of 142 subjects including healthy controls (HCs), patients with CHB, liver cirrhosis (LC), liver cirrhosis-acute decompensation (LC-AD) and ACLF. All HCs, CHB and LC subjects were recruited from the outpatient clinic. Among the 38 enrolled ACLF patients, 21 survived patients were placed into ACLF-S group while the remaining 17 were placed into ACLF-D group. Patients with ACLF received a standard treatment, including nucleoside analogs for HBV DNA-positive patients; diammonium glycyrrhizinate and Ademetionine for protection of liver function; sodium restriction, diuretics and paracentesis combined with albumin infusion for ascites; lactulose and L-ornithine aspartate for HE; prophylactic antibiotics for bacterial infections and renal replacement therapy for hepato-renal syndrome. Eleven non-survivors (11/38) and 35 survivors (35/124) received plasma exchange (PE) combined with hemofiltration (HF) treatment. All subjects were enrolled or recruited in the First Affiliated Hospital of Zhejiang University. This study was conducted in compliance with the principles of the Declaration of Helsinki and was approved by the Ethics Committee of the First Affiliated Hospital of Zhejiang University. Written informed consent was obtained from all patients or their legal representatives.

### Inclusion and Exclusion Criteria

ACLF was diagnosed according to the Asian Pacific Association for the Study of the Liver (APASL) criteria published in 2014 (3). According to the APASL criteria, ACLF was defined as an acute hepatic insult manifesting as jaundice (serum levels of bilirubin ≥ 5 mg/dl) and coagulopathy (international normalized ratio [INR] ≥ 1.5 or prothrombin activity <40%) within 4 weeks in a patient with previously diagnosed or undiagnosed CLD/cirrhosis. Cirrhosis patients with a history of AD who met the above criteria were also diagnosed as ACLF. The Model for End-stage Liver Disease (MELD), the chronic liver failure-sequential organ failure assessment (CLIF-SOFA), CLIF-C ACLF (18) scores were calculated to evaluate the severity of patients. The diagnosis of LC was based on previous liver biopsy, endoscopic signs, radiologic imaging, clinical symptoms and laboratory parameters. HBV reactivation was defined as a ≥ 2 log increase in the HBV DNA level from previously stable baseline level or a level ≥ 100 IU/mL in patients in whom HBV DNA had been undetectable, or ≥ 20,000 IU/mL in those negative for HBV DNA at baseline (19). The exclusion criteria included: age <18 or >80 years, liver malignancy or any other type of cancer, history of liver transplantation or bone marrow transplantation, and any severe disease in other systems.

### Data Collection

Data were collected at enrollment, and included vital signs, physical examination results, histories, complications and precipitating events. The laboratory measurements collected included white blood cell (WBC) count, neutrophils, red blood cell (RBC) count, platelet (PLT) count, C-reactive protein (CRP), alpha-fetoprotein (AFP), ferritin, total protein (TP), alanine aminotransferase (ALT), aspartate aminotransferase (AST), serum levels of albumin (ALB), cholinesterase, gamma-glutamyl transpeptidase (GGT), triglycerides, cholesterol, total bilirubin (TB) level, serum levels of sodium, potassium (K), creatinine (Cr), blood urea nitrogen (BUN), international normalized ratio (INR), pulse oximetry, HBV infection biomarker levels, and HBV-DNA levels. Image measurements, including ultrasound, CT, and MRI, were also collected. Follow-up information was collected from medical records and follow-up phone calls at 30 days.

### Sample Collection and Testing

Blood samples were obtained on the day following hospitalization and were centrifuged at 3,500 rpm for 10 min to separate the serum, which were stored at −80°C. GH was measured using an enzyme-linked immunosorbent assay (ELISA) with an ELISA detection kit (ab190811, Abcam). IGF-1, IGF-2, IGF receptor-1, and IGF receptor-2 levels in prospective cohort were detected using an immunoassay (QAH-IGF-1, RayBiotech). IGF-1 level in cross-sectional cohort was detected using an ELISA detection kit (ab211651, Abcam).

### Statistical Analyses

Statistical analyses were performed using Statistical Package for the Social Sciences (SPSS, v.22.0; SPSS, Inc., Chicago, IL, USA) and MEDCALC (MedCalc Software, Belgium). Continuous data are expressed as means ± standard deviations or medians with interquartile ranges (p25, p75), while categorical data are expressed as numbers (percentages). All tests were two-tailed, and *P* < 0.05 were considered indicative of statistical significance. Student's *t*-tests or non-parametric Mann–Whitney *U*-tests, as appropriate, were used to compare continuous data. Categorical data and ordered categorical data were compared using chi-square tests and Spearman rank correlation tests, respectively. The area under the receiver operating characteristic curve (AUROC) of the various prognostic scoring systems was compared by Z-tests using Delong's method.

## Results

### Characteristics of the Prospective HBV-ACLF Cohort

A total of 124 HBV-ACLF patients were enrolled in the prospective cohort. As shown in Table 1, the mean patient age was 46.61 ± 12.71 years, and 19 (15.3%) patients were female. Among the whole cohort, 69 (55.6%) patients were cirrhotic, and 25 (20.2%) had acute decompensation (AD) history. The MELD, CLIF-SOFA, CLIF-C ACLF scores were 23.09 ± 5.61, 8.15 ± 1.95, and 42.01 ± 9.79, respectively.

**Table 1:** Characteristics of non-survivors and survivors in the HBV-ACLF cohort.

	**Total (*n =* 124)**	**Non-survivor (*n =* 38)**	**Survivor (*n =* 86)**	***p*-value (non-survivor vs. survivor)**
Female, n(%)	19 (15.3)	8 (21.1)	11 (12.8)	0.239
Age (y)	46.61 ± 12.71	51.21 ± 12.9	44.58 ± 12.15	0.009
MAP (mm Hg)	89.86 ± 12.77	90.11 ± 17.16	89.75 ± 10.38	0.903
Alcoholism	13 (10.5)	7(18.4)	6 (7.0)	0.055
Cirrhosis	69 (55.6)	17 (44.7)	52 (60.5)	0.104
Previous decompensation	25 (20.2)	4 (10.5)	21 (24.4)	0.075
GIB	13 (10.5)	4 (10.5)	9 (10.5)	0.608
Infection	38 (30.6)	14 (36.8)	24 (27.9)	0.32
Ascites	74 (59.7)	24 (63.2)	50 (58.2)	0.599
HE	19 (15.3)	16 (42.1)	3 (3.5)	<0.0001
HBeAg-positive	50 (40.3)	14 (36.8)	36 (41.9)	0.599
LgDNA	5.37 ± 2.02	5.27 ± 2.25	5.41 ± 1.93	0.724
WBC (×10^9^/L)	7.05 (5.4, 9.88)	8.45 (5.75, 11.55)	6.7 (4.98, 8.9)	0.011
NEU (×10^9^/L)	5.1 (3.43, 6.98)	6.4 (4.23, 10.2)	4.65 (3.28, 6.15)	0.005
RBC (×10^9^/L)	4.15 (3.68, 4.63)	4.19 (3.69, 4.65)	4.13 (3.68, 4.6)	0.856
PLT (×10^9^/L)	113.4 ± 49.92	110.29 ± 48.22	114.77 ± 50.87	0.647
GH (pg/ml)	2,232 (1176.9, 4300.88)	1106.55 (674.25, 1922.4)	2930.55 (1778.85, 4799.33)	<0.0001
Ferritin (ng/ml)	2567.95 (1170.2, 4166.35)	2459.45 (1021.25, 5021.18)	2567.95 (1262.83, 3766.18)	0.704
Alpha-fetoprotein (ng/ml)	117.9 (40.48, 294.48)	111.85 (39.63, 277.6)	124.1 (40.48, 320.28)	0.615
CRP (mg/L)	11.15 (8.28, 15.45)	11.75 (9.3,15.78)	10.65 (8.1, 15.5)	0.191
TP (g/L)	58.15 (53.53, 62.38)	58.2 (54.03, 61.35)	58.1 (53.3, 62.55)	0.901
ALT (U/L)	268 (107.5, 596.25)	281 (140.75, 430.5)	257.5 (84, 700.75)	0.67
AST (U/L)	162.5 (86.5, 367)	178.5 (111, 401.75)	162 (85, 336.25)	0.265
ALB (g/L)	31.27 ± 4.26	30.06 ± 4.55	31.81 ± 4.03	0.035
ALP (U/L)	146.02 ± 39.74	148.05 ± 40.01	145.12 ± 39.82	0.706
Cholinesterase (U/L)	3721.79 ± 1438.38	3453.11 ± 1469.41	3840.51 ± 1416.85	0.168
GGT (U/L)	102.25 ± 58.9	93.03 ± 56.19	106.33 ± 59.93	0.248
Total Bilirubin (mmol/L)	299.65 (232.03, 405.3)	354.5 (288.73, 454.1)	278.15 (213.75, 381.78)	0.001
Creatine (mmol/L)	64 (57, 73.75)	72 (57, 115.25)	63 (57, 69.25)	0.005
BUN (mmol/L)	4.06 (3.11, 6.16)	5.28 (3.64, 7.74)	3.75 (3.05, 5.52)	0.003
Triglycerides (mmol/L)	1.28 (1.02, 1.66)	1.17 (0.98, 1.41)	1.35 (1.03, 1.79)	0.039
Cholesterol (mmol/L)	2.22 (1.69, 2.76)	1.7 (1.48, 2.62)	2.38 (1.84, 2.79)	0.003
Potassium (mmol/L)	4.22 (3.9, 4.78)	4.39 (3.94, 4.95)	4.16 (3.87, 4.6)	0.08
Sodium (mmol/L)	138 (135.25, 139)	137.5 (134.75, 139.25)	138 (136, 139)	0.703
Blood glucose (mmol/L)	4.05 (3.21, 5.1)	3.85 (2.96, 5.67)	4.12 (3.25, 4.92)	0.612
INR	1.99 (1.64, 2.53)	2.69 (2.32, 3.28)	1.86 (1.59, 2.19)	<0.0001
MELD	23.09 ± 5.61	28.44 ± 5.74	20.73 ± 3.57	<0.0001
CLIF-SOFA	8.15 ± 1.95	10 ± 2.41	7.33 ± 1.14	<0.0001
CLIF-C ACLF	42.01 ± 9.79	50.49 ± 9.69	38.27 ± 7.18	<0.0001

*Continuous data are expressed as means ± SD or medians with interquartile ranges (p. 25, p. 75), and categorical data are expressed as numbers (percentages). ALP, phosphatase alkaline; ALT, alanine aminotransferase; AST, aspartate aminotransferase; BUN, blood urea nitrogen; CLIF-C ACLF, European Association for the Study of Chronic Liver Failure; COSSH-ACLF, Chinese Group on the Study of Severe Hepatitis B; CRP, C-reactive protein; GGT, gamma-glutamyl transpeptidase; HBV, hepatitis B virus; HE, hepatic encephalopathy; INR, international normalized ratio; MAP, mean arterial pressure; MELD, Model for End-stage Liver Disease; RBC, red blood count; GIB, gastrointestinal hemorrhage; WBC, white blood count*.

Based on 30-day survival, the patients were divided into non-survivor (*n* = 38) and survivor groups (*n* = 86). The mean age of survivor and non-survivor groups was 44.58 ± 12.15 and 51.21 ± 12.9, while 12.8% in survivor group were female and 21.1% in non-survivor group. Compared to the survivor group, non-survivors were older (*P* = 0.009) and had lower levels of cholesterol (*P* = 0.003) and albumin (*P* = 0.035). Conversely, neutrophil counts (*P* = 0.005), INR (*P* < 0.0001), TB (*P* = 0.001) and creatine (*P* = 0.005) levels, and MELD (*P* < 0.0001), CLIF-SOFA (*P* < 0.0001), and CLIF-C ACLF (*P* < 0.0001) scores were higher in the non-survivor group.

### Distribution of GH in Prospective Cohort Patients

**Figure 2:**
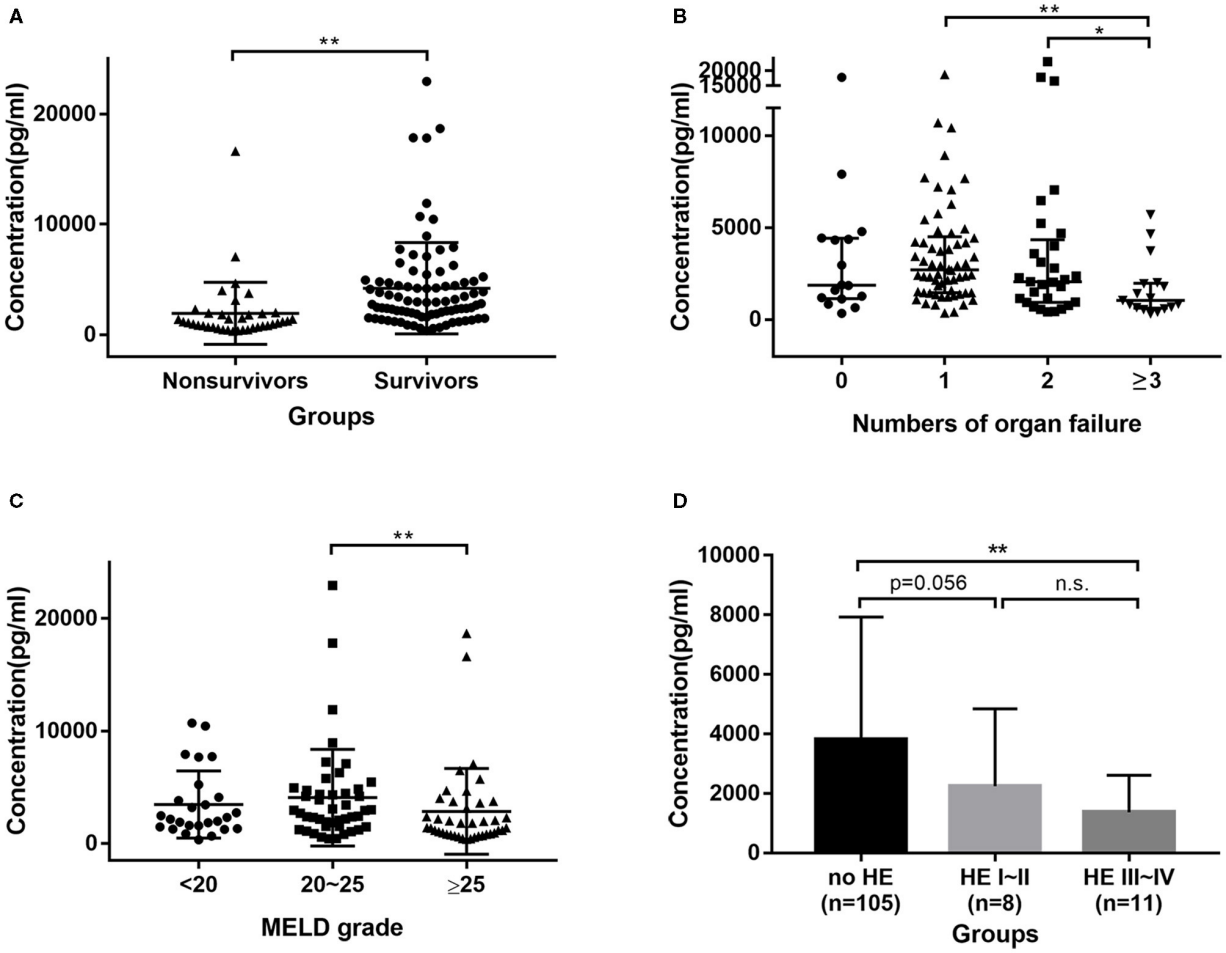
Distribution of GH in HBV-ACLF cohort. **(A)** GH levels were higher in survivors group than in non-survivors group. **(B)** Patients with 3 or more organs failure had lower GH levels than patients with 2, 1, or no organ failure. **(C)** Patients with MELD > 25 had lower GH levels than patients with MELD between 20 and 25. **(D)** patients with Grade III~IV encephalopathy had lower GH levels than no HE group. * <0.05, ** <0.01.

In the prospective cohort of HBV-ACLF patients, the median GH levels in non-survivors were 1106.55 pg/ml, which were significantly lower (*P* < 0.0001) than in survivors (2930.55 pg/ml) (Figure 2A). All patients were divided into four groups according the number of organ failures; patients with ≥3 organ failures had significantly lower GH levels compared to 1 (*P* < 0.001), and 2 (*P* = 0.045) organ failure groups (Figure 2B). Furthermore, patients with a MELD score > 25 had lower GH levels than those with a score between 20 and 25 (*P* = 0.009) (Figure 2C). However, there were no significant difference in GH levels between the MELD > 25 group and the MELD <20 group. Comparisons of GH levels between patients with and without certain kinds of organ failure are shown in Supplementary Figure 1. Interestingly, patients with encephalopathy had lower GH levels than those without. We divided patients into no HE, HE I~II, HE III~IV groups (Figure 2D), and then found that GH level in no HE group was significantly higher than HE III~IV group (*P* = 0.002), and showed a higher trend than HE I~II group (*P* = 0.056).

The relationships between GH levels and other indicators are shown in Supplementary Table 1. GH levels were negatively related to neutrophil counts, CRP, and BUN, but positively related to ALB, triglycerides, cholesterol, and alkaline phosphatase. The serum levels of IGF-1, IGF-2, IGF-1R, and IGF-2R were measured, as shown in Supplementary Table 2, but there were no significant differences between survivors and non-survivors.

### GH Levels in the Cross-Sectional Cohort

**Figure 3:**
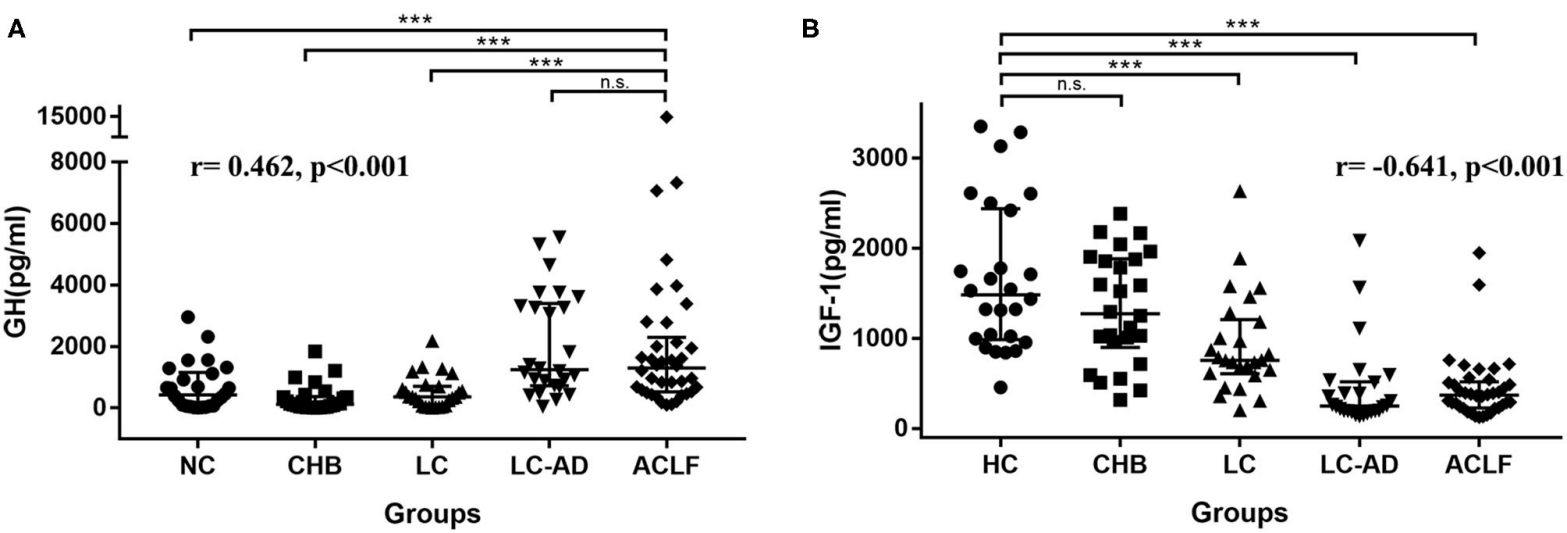
Distribution of GH in cross-sectional cohort. **(A)** ACLF group showed higher GH levels than HC, CHB, and LC group, but no difference from LC-AD group, and the *r*-value of the correlation between GH levels and severity was 0.462, *p* < 0.001. **(B)** HC group showed higher IGF-1 levels than LC, LC-AD, and ACLF group, but no difference from CHB group, and the r value of the correlation between IGF-1 levels and severity was −0.641, *p* < 0.001. ACLF, acute-on-chronic liver failure; CHB, chronic hepatitis B; GH, growth hormone; HC, health control; IGF-1, insulin-like growth factor-1; LC, liver cirrhosis; LC-AD, liver cirrhosis-acute decompensation. *** < 0.001.

The characteristics of HCs and patients in the cross-sectional cohort are listed in Table 2. There were no differences in mean age or sex among groups. The median GH levels in each group were 427.66 pg/ml (HCs), 125.91 pg/ml (CHB), 362.94 pg/ml (LC), 1246.05 pg/ml (LC-AD), and 1299.8 pg/ml (ACLF). Comparisons of GH levels among the different groups are shown in Figure 3A. Compared to HC (*P* < 0.001), CHB (*P* < 0.001), and LC (*P* < 0.001), the ACLF group had significantly higher GH levels. Similarly, patients in the LC-AD group had higher GH levels than those in the HC (*P* < 0.001), CHB (*P* < 0.001), and LC (*P* < 0.001) groups. In ACLF group, ACLF-S group had higher GH level than ACLF-D group, which was shown in Supplementary Figure 2. A detailed comparison of GH levels among groups is listed in Supplementary Table 3. Correlation analysis revealed a positive correlation between GH levels and disease severity (*r* = 0.462, *P* < 0.001).

**Table 2:** Characteristics of the cross-sectional cohort.

	**HC (*n =* 26)**	**CHB (*n =* 26)**	**LC (*n =* 26)**	**LC-AD (*n =* 26)**	**ACLF group**		
					**Overall (*n =* 38)**	**ACLF-S (*n =* 21)**	**ACLF-D (*n =* 17)**
Age	42.42 ± 12.88	43.08 ± 12.94	48.81 ± 8.07	52.62 ± 13.12	49.1 ± 13.3	51.81 ± 13.47	45.65 ± 12.57
Sex (M/F)	11/15	16/10	20/6	20/6	32/6	17/4	15/2
WBC (×10^9^/L)	5.85 (4.98, 7.03)	5.25 (3.73, 6.23)	4 (3.1, 5)	3.5 (2.13, 4.9)	7.2 (5.8, 8.6)	6.1 (4.9, 7.9)	7.8 (6.8, 9.9)
RBC (×10^9^/L)	4.65 (4.35, 4.83)	4.33 (4.14, 4.64)	4.64 (4.12, 5.22)	3.43 (2.66, 3.79)	4.2 (3.6, 4.5)	4.06 (3.53, 4.33)	4.41 (4.01, 4.68)
Platelets (×10^9^/L)	243.5 (203.25, 300)	190.5 (132.25, 245.5)	108 (65, 160)	52.5 (40, 92.75)	100.5 (73.5, 145.5)	93 (72, 138.5)	123 (69, 146.5)
TP (g/L)	73.7 (70.25, 76.13)	68.5 (62.8, 76.2)	70.4 (64, 76.35)	57.3 (53.08, 61.88)	57.8 (54, 62.7)	58.5 (54.5, 65.75)	55.8 (53.15, 61.65)
Albumin (g/L)	47.35 (45.28, 49.93)	41.6 (39.4, 44.5)	41.3 (36.4, 46)	28.9 (24.1, 34.1)	29.9 (27.8, 33.5)	29.8 (27.15, 33.55)	30.9 (27.9, 33.35)
ALT (U/L)	13.5 (10, 23)	47.5 (28.25, 89)	28.5 (22.5, 53.75)	27.5 (16.5, 90.5)	294.5 (158.3, 598.5)	248 (144, 404.5)	375 (213.5, 766)
AST (U/L)	17 (14, 20.5)	36 (21.5, 59)	29.5 (23.75, 42.5)	49.5 (24.75, 96)	294.5 (164.8, 409.8)	287 (127, 486)	306 (209.5, 376.5)
Total bilirubin (mmol/L)	11.5 (8, 14)	12.5 (8.75, 18.5)	12 (11,17.25)	40.5 (31.5, 76)	355.5 (226, 407)	292 (188.5, 384)	391 (301, 452.5)
Creatinine (mmol/L)	67.5 (56.75, 80)	73 (65, 85.5)	74.5 (64.5, 82.75)	79 (65.75, 91.25)	66 (55.5, 76.3)	68 (62, 75)	58 (52.5, 83)
BUN (mmol/L)	5.15 (4.25, 6.2)	5 (3.85, 6.45)	5.67 (4.55, 6.36)	5.6 (4.02, 7.78)	3.8 (2.8, 5.2)	3.8 (3.15, 4.25)	3.5 (2.15, 5.7)
INR	ND	ND	1.14 (1.07, 1.22)	1.45 (1.34, 1.58)	2.4 (1.9, 2.8)	2.1 (1.86, 2.37)	2.73 (2.47, 4)
GH (pg/ml)	427.66 (64.27, 1162.01)	125.91 (38.46, 373.35)	362.94 (69.27, 709.34)	1246.05 (723.66, 3397.5)	1299.8 (528.2, 2302.3)	1616.62 (843.18, 3101.43)	680.71 (398.71, 1451.91)

*Continuous data are expressed as means ± SD or medians with interquartile ranges (p. 25, p. 75), and categorical data are expressed as numbers (percentages). ALT, alanine aminotransferase; AST, aspartate aminotransferase; BUN, blood urea nitrogen; CHB, chronic Hepatitis B; GH, growth hormone; INR, international normalized ratio; LC, liver cirrhosis; LC-AD, liver cirrhosis-acute decompensation; ND, not detected; RBC, red blood count; TP, total protein; WBC, white blood count*.

Distribution of IGF-1 among these groups was different from GH. As shown in Figure 3B, IGF-1 levels in HC group were higher than LC (*P* < 0.001), LC-AD (*P* < 0.001) and ACLF (*P* < 0.001) group. Interestingly, IGF-1 levels in LC-AD group were significantly lower than in LC group (*P* < 0.001), but GH levels revealed much higher (*P* < 0.001). Consistent with the result in prospective cohort, there was no difference of IGF-1 levels between ACLF-S and ACLF-D groups. A detailed comparison of IGF-1 levels among groups is listed in Supplementary Table 4. Correlation analysis revealed a negative correlation between IGF-1 levels and disease severity (*r* = −0.641, *P* < 0.001).

### Roles of GH in Predicting the Outcome of HBV-ACLF Patients

Univariate analyses showed that age, HE, GH, WBC, neutrophil count, ALB, TB, Cr, BUN, cholesterol, and INR were significantly associated with 30-day outcomes of HBV-ACLF patients in the prospective cohort. Then, multivariate analyses revealed that HE, GH, WBC, TB, BUN, and INR were independently associated with prognosis at day 30 (Table 3).

**Table 3:** Univariate and multivariate logistic regression analyses of 30-day survival in the study cohort.

**Variables**	**Univariate logistic regression**			**Multivariate logistic regression**		
	**HR**	**95%CI**	***p*-value**	**HR**	**95%CI**	***p*-value**
Age	0.958	[0.928, 0.989]	**0.009**			
Sex	1.818	[0.666, 4.964]	0.243			
MAP	0.998	[0.968, 1.028]	0.883			
Cirrhosis	1.889	[0.873, 4.088]	0.106			
Previous decompensation	2.746	[0.872, 8.646]	0.084			
Alcoholism	0.064	[0.103, 1.067]	0.064			
Bacterial infection	0.664	[0.295, 1.492]	0.321			
Acites	0.810	[0.369, 1.778]	0.600			
UGIB	0.994	[0.286, 3.450]	0.992			
HE	0.050	[0.013, 0.186]	** <0.0001**	0.03	[0.003, 0.275]	**0.002**
Growth hormone (mg/dL)	1.436	[1.122, 1.838]	**0.004**	1.708	[1.113, 2.619]	**0.014**
WBC(×109/L)	0.885	[0.800, 0.979]	**0.018**	1.345	[1.053, 1.718]	**0.017**
Neutrophil (×10^9^/L)	0.861	[0.769, 0.964]	**0.010**			
RBC (×10^9^/L)	1.030	[0.897, 1.181]	0.678			
Platelets (×10^9^/L)	1.002	[0.994, 1.010]	0.644			
Ferritin (ng/ml)	0.942	[0.825, 1.076]	0.378			
Alpha-fetoprotein (ng/ml)	1.001	[0.999, 1.002]	0.333			
CRP (mg/L)	0.983	[0.949, 1.018]	0.337			
Total protein (g/L)	0.996	[0.951, 1.043]	0.878			
Albumin (g/L)	1.108	[1.006, 1.220]	**0.038**			
ALT (U/L)	1.000	[0.999, 1.001]	0.619			
AST (U/L)	0.999	[0.998, 1.000]	0.163			
ALP (U/L)	0.998	[0.989, 1.008]	0.703			
Cholinesterase (U/L)	0.169	[0.919, 1.620]	0.169			
Total bilirubin (mmol/L)	0.995	[0.991, 0.998]	**0.001**	0.987	[0.979, 0.996]	**0.002**
Cr (mmol/L)	0.968	[0.949, 0.987]	**0.001**			
BUN (mmol/L)	0.761	[0.647, 0.897]	**0.001**	0.566	[0.415, 0.772]	**0.0002**
Triglycerides (mmol/L)	2.385	[0.996, 5.709]	0.051			
Cholesterol (mmol/L)	2.163	[1.182, 3.957]	**0.012**			
Potassium (mmol/L)	0.543	[0.276, 1.069]	0.077			
Sodium (mmol/L)	1.024	[0.924, 1.136]	0.648			
Blood glucose (mmol/L)	0.208	[0.823, 1.043]	0.927			
INR	0.136	[0.060, 0.306]	** <0.0001**	0.037	[0.007, 0.214]	** <0.0001**

*ALP, phosphatase alkaline; ALT, alanine aminotransferase; AST, aspartate aminotransferase; BUN, blood urea nitrogen; CRP, C-reactive protein; GGT, gamma-glutamyl transpeptidase; HE, hepatic encephalopathy; INR, international normalized ratio; MAP, mean arterial pressure; RBC, red blood count; UGIB, upper gastrointestinal hemorrhage; WBC, white blood count. The bold value mean the p < 0.05*.

**Figure 4:**
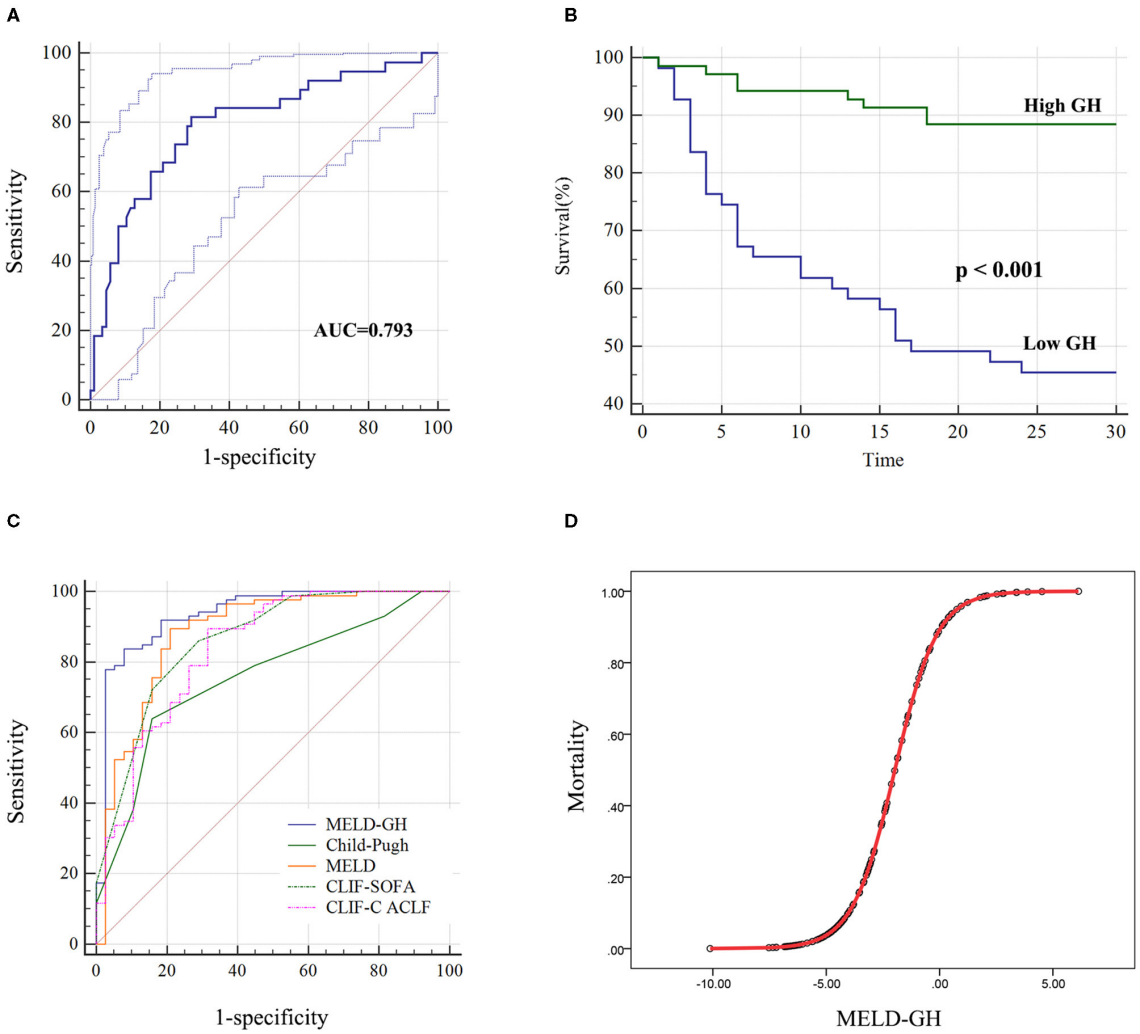
Performance of GH on predicting 30-day survival of HBV-ACLF patients. **(A)** The AUC of GH predicting the outcome was 0.793, and low GH (<2,001 pg/ml) group **(B)** showed significantly worse 30-day outcome than high GH group. **(C)** MELD-GH score showed highest AUC than Child-Pugh, MELD, CLIF-SOFA, and CLIF-C ACLF scores. **(D)** The estimation of HBV-ACLF mortality based on MELD-GH score.

Next, we analyzed the prognostic value of GH. The AUC of GH for predicting 30-day outcomes was 0.793 (Figure 4A). Then we divided patients into low-GH and high-GH groups according to the cut-off value (2,001 pg/mL). The 30-day survival rate in low-GH group was significantly lower than in the high-GH group (45.5 vs. 88.4%, *P* < 0.001) (Figure 4B).

Then we analyzed the prognostic efficacy among different prognostic models. The AUC of Child-Pugh, MELD, CLIF-SOFA and CLIF-C ACLF was 0.758, 0.882, 0.862, and 0.838. A new prognostic model was built based on MELD score by logistic regression analyses, namely MELD-GH, combining GH with MELD to predict 30-day outcome. The formula used to calculate MELD-GH is: 0.413^*^MELD-1.606^*^Ln (GH). The sensitivity and specificity of MELD-GH was 83.72% and 92.11%. The AUC of MELD-GH was 0.937, which was significantly superior to the Child-Pugh (*P* < 0.0001), MELD (*P* = 0.0185), CLIF-SOFA (*P* = 0.034), and CLIF-C ACLF (*P* = 0.0227) scores for predicting 30-day outcomes (Figure 4C and Table 4). When MELD-GH < −5, the mortality rate only reached 3.5%, and when MELD-GH > 0, the mortality rate increased above 90% (Figure 4D). The detailed comparison of each two prognostic models were listed in Supplementary Table 5.

**Table 4:** Performance of different prognostic models.

**Variables**	**Sensitivity (%)**	**Specificity (%)**	**AUC**	**SE**	**95% CI**	***p*-value (*vs*. MELD-GH)**
MELD-GH	83.72	92.11	0.937	0.026	0.878–0.973	
Child-pugh	63.95	84.21	0.758	0.045	0.673–0.830	<0.0001
MELD	89.53	78.95	0.882	0.037	0.812–0.933	0.0185
CLIF-SOFA	86.09	71.05	0.862	0.035	0.788–0.917	0.0344
CLIF-C ACLF	89.53	68.42	0.838	0.042	0.761–0.898	0.0227

*CLIF-C ACLF, European Association for the Study of Chronic Liver Failure; CLIF-SOFA, chronic liver failure-sequential organ failure assessment; COSSH-ACLF, Chinese Group on the Study of Severe Hepatitis B; MELD, Model for End-stage Liver Disease*.

## Discussion

We analyzed serum levels of GH between non-surviving and surviving HBV-ACLF patients. Low GH levels predicted poor outcomes in HBV-ACLF patients. GH is bound by the GH receptor on the cell membrane of hepatocytes, where it activates the Janus kinase 2 (JAK2)-signal transducer and activator of transcription 5 (STAT5) signaling pathway to upregulate genes such as IGF-1 and peroxisome proliferator-activated receptor γ (PPAR-γ) and thereby modulate lipid metabolism (13). IGF-1 is mainly synthesized in the liver under the regulation of GH, which further affects energy metabolism in hepatocytes. In addition, GH can promote the regeneration of hepatocytes (8, 20). In the current study, GH levels were significantly higher in the ACLF-S group than in the ACLF-D group, possibly because high concentrations of GH maintained the metabolic function or promoted the regeneration of hepatocytes.

The liver is the major source of circulating IGF-1, and its bioavailability is modified by insulin-like growth factor binding proteins (IGFBPs) (11). We measured the concentrations of IGF-1 and IGF-2, but there were no differences between the survivor and non-survivor groups. A possible explanation for this is that HBV-ACLF patients suffered GH resistance, which was defined by high levels of circulating GH and low levels of IGF (21). GH resistance is often observed in cirrhotic patients, who develop nutritional and metabolic complications such as insulin resistance, malnutrition, osteopenia, and hypogonadism, which is in part related to IGF-1 deficiency (17). In the current study, GH levels were increased in the survival group, but IGF-1 levels were not increased significantly due to GH resistance. Whether GH can upregulate the IGF-1 level remains uncertain, and how GH exerts its effects in hepatocytes remains unclear.

In the cross-sectional cohort, GH concentrations were higher in the LC-AD group than in the LC group, suggesting that elevated GH levels may be associated with acute insult of decompensation. In children with bacterial sepsis and septic shock, GH levels were elevated significantly, which is in contrast to the changes observed in IGF-1 levels (22). In addition, Wang et al. (23) demonstrated that inhibition of the GH pathway aggravates acetaminophen-induced acute mice liver injury. Therefore, when an acute insult occurs, GH secretion is increased in response to the stress. In our cross-sectional study, we observed that IGF-1 decreased as the severity of the HBV disease progressed. LC group had lower IGF-1 levels than HC and CHB groups, which is consistent with other studies (24, 25). Interestingly, in the LC-AD and ACLF groups, IGF-1 levels were much lower than LC group, which revealed that the synthesis function of hepatocytes was seriously damaged. However, IGF-1 level didn't decreased further in ACLF group than LC-AD group and was not affected by the increase of GH level.

The number of organ failures determines the mortality rate of patients with ACLF (26). Our study found that when patients had two or more organ failures, the concentration of GH dropped dramatically. This suggests that GH secretion may be closely related to the general condition of the patient. In the comparison of different organ failures, GH levels were significantly lower in the brain failure group compared to the non-brain failure group, which suggests that GH secretion is affected by encephalopathy. Liu et al. (27) found that IGF-1 and GH levels decreased as the severity of encephalopathy worsened, which is consistent with the current results. However, due to the small number of patients with encephalopathy, this result needs to be confirmed further.

In this study, we developed a new prognostic model, namely MELD-GH, which was based on the MELD score. Further analyses demonstrated a better prognostic efficacy of MELD-GH than Child-Pugh, MELD, CLIF-SOFA, and CLIF-C ACLF scores. This result suggested GH may be applied as one of the indicators for predicting the short-term outcome of HBV-ACLF patients. MELD-GH had better prognostic efficacy was significantly more convenient to calculate and apply in the clinical setting, because it only contained 4 indicators, which was less than CLIF-ACLF and CLIF-C ACLF. In our study, we used APASL criteria to diagnose ACLF and used CLIF-SOFA and CLIF-C ACLF to evaluated the severity and make a comparison with the new model. We used APASL consensus criteria to diagnose ACLF because our cohort characteristics were more similar to the Asian ACLF, which was mainly caused by HBV (3). Though CLIF-SOFA and CLIF-C-ACLF score were built according to the ACLF patients with decompensated cirrhosis, they were widely recognized and usually used for comparison with new models (28–30).

In a previous study, thyroid-related hormones also differed in patients with ACLF (31). Interestingly, thyroid-stimulating hormone can predict the prognosis of patients with ACLF. In addition, some studies have found that sex hormones (32) and adrenal hormones (33) have prognostic value in cirrhosis or liver failure. It could be speculated that endocrine organs, particularly the pituitary gland, may play an important role in the pathogenesis of ACLF. Some other studies have shown that pituitary function changes before and after liver transplantation (20, 34). and may be related to disease severity and liver regeneration.

There were some limitations to this study. First, the mechanism by which GH participates in the pathophysiology of the ACLF remains elusive. Without any change in IGF-1, the mechanism of the physiological role of GH and downstream regulatory molecules in the GH pathway remain elusive, which limits our observations regarding the GH-mediated regulation of liver regeneration and metabolism. Second, we only enrolled HBV-ACLF patients, whether these results can be applied to other causes of ACLF remains to be confirmed. Third, the prognostic efficacy of GH and MELD-GH needs to be validated in a large multicenter study.

## Conclusions

In our HBV-ACLF cohort, serum levels of GH were higher in the survival group and the high-GH patients also had longer survival than who with low-GH, which suggested that low levels predicted poor outcomes in HBV-ACLF patients. Low levels predicted poor outcomes in HBV-ACLF patients. MELD-GH scores had better predictive accuracy than Child-Pugh, MELD, CLIF-SOFA, and CLIF-C ACLF scores. Pituitary function might play a role in ACLF, which needs further research.

## Data Availability Statement

The raw data supporting the conclusions of this article will be made available by the authors, without undue reservation.

## Ethics Statement

The studies involving human participants were reviewed and approved by Research Ethics Committee of the First Affiliated Hospital, College of Medicine, Zhejiang University. Written informed consent to participate in this study was provided by the participants' legal guardian/next of kin.

## Author Contributions

XX, ZX, and DW: study concept and design. LZ, SM, YZ, and FZ: data acquisition. ZX, DW, and RC: data analysis and interpretation. XX and ZX: critical revision of the manuscript for important intellectual content. ZX and QL: statistical analysis. XX and DW: obtained funding. All authors reviewed and approved the final manuscript.

## Conflict of Interest

The authors declare that the research was conducted in the absence of any commercial or financial relationships that could be construed as a potential conflict of interest.
