# Supplementary material for: Low Growth Hormone Levels Predict Poor Outcome of Hepatitis B Virus-Related Acute-on-Chronic Liver Failure
Source: Front Med (Lausanne). 2021 Jul 6;8:655863. doi: 10.3389/fmed.2021.655863 (PMC8290074; doi:10.3389/fmed.2021.655863)
Supplement: Supplementary file 1 [file Data_Sheet_1.DOCX]

**Supplementary materials**


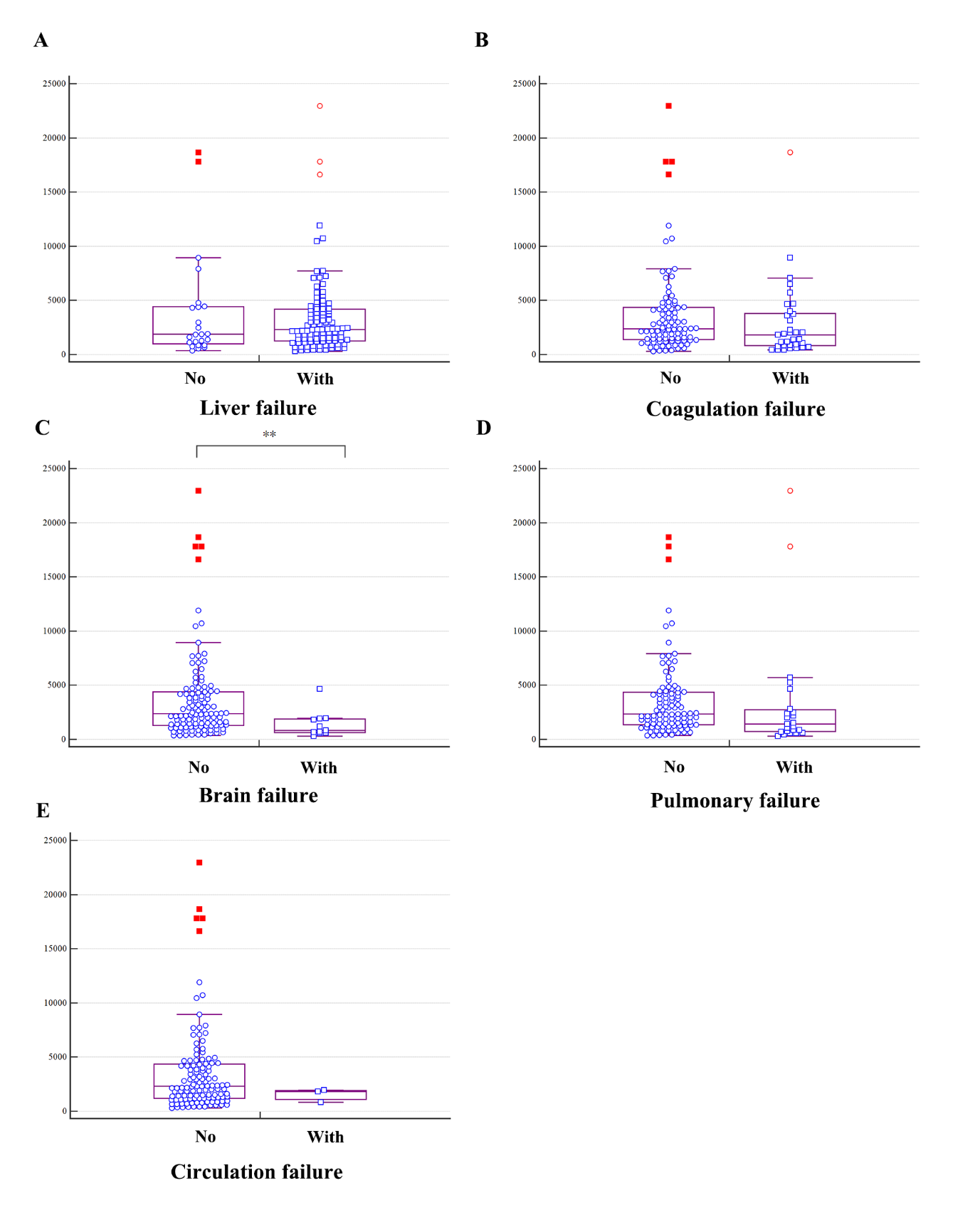


Supplementary Figure 1 Distribution of GH levels in different organ failure groups. **<0.01


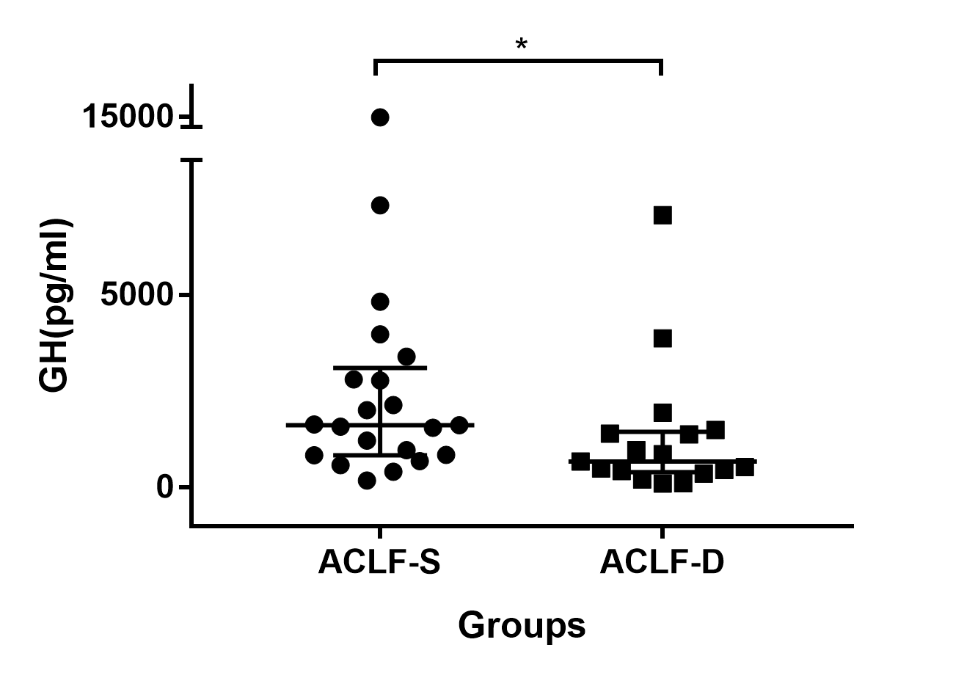


Supplementary Figure 2 Distribution of GH between ACLF-S and ACLF-D groups in the cross-sectional cohort.*p<0.05.

Supplementary Table 1 Coeffecients between GH and other indicators

|  | coeffecients | p value |
| --- | --- | --- |
| White blood cells | -0.259 | 0.004 |
| C-reaction protein | -0.213 | 0.018 |
| Neutrophil | -0.258 | 0.004 |
| Albumin | 0.208 | 0.02 |
| Alkaline phosphatase | 0.182 | 0.043 |
| Blood urea nitrogen | -0.277 | 0.002 |
| Triglycerides | 0.184 | 0.041 |
| Cholesterol | 0.192 | 0.032 |

Supplementary Table 2 Serum level of IGF-1, IGF-2, IGF-1R and IGF-2R between survivors and nonsurvivors

|  | Nonsurvivors | Survivors | p value |
| --- | --- | --- | --- |
| IGF-1 (pg/ml) | 213.41(76.55,290.53) | 206.68(81.14,390.46) | 0.408 |
| IGF-2 (pg/ml) | 56.39(24.36,116.04) | 75.56(41.45,133.61) | 0.301 |
| IGF-1R (pg/ml) | 810.41(469.85,996.91) | 793.89(464.86,1210.18) | 0.751 |
| IGF-2R (pg/ml) | 199.74(96.85,283) | 197.57(149.51,290.38) | 0.352 |

Supplementary Table 3 p values of comparison of GH levels between different groups in cross-sectional cohort

|  | HC | CHB | LC | LC-AD | ACLF-S |
| --- | --- | --- | --- | --- | --- |
| CHB | 0.063 |  |  |  |  |
| LC | 0.621 | 0.076 |  |  |  |
| LC-AD | **<0.001** | **<0.001** | **<0.001** |  |  |
| Total ACLF | **<0.001** | **<0.001** | **<0.001** | 0.503 |  |
| ACLF-S | **<0.001** | **<0.001** | **<0.001** | 0.669 |  |
| ACLF-D | **0.091** | **<0.001** | **0.024** | 0.087 | **0.017** |

Supplementary Table 4 p values of comparison of IGF-1 levels between different groups in cross-sectional cohort

|  | HC | CHB | LC | LC-AD | ACLF-S |
| --- | --- | --- | --- | --- | --- |
| CHB | 0.297 |  |  |  |  |
| LC | **<0.001** | **0.012** |  |  |  |
| LC-AD | **<0.001** | **<0.001** | **<0.001** |  |  |
| Total ACLF | **<0.001** | **<0.001** | **<0.001** | 0.169 |  |
| ACLF-S | **<0.001** | **<0.001** | **<0.001** | 0.32 |  |
| ACLF-D | **<0.001** | **<0.001** | **0.003** | 0.18 | 0.607 |

Supplementary Table 5 p values of comparison of AUC of different prognostic models

|  | MELD-GH | Child-Pugh | MELD | CLIF-SOFA |
| --- | --- | --- | --- | --- |
| Child-Pugh | **<0.0001** |  |  |  |
| MELD | **0.0185** | **0.0067** |  |  |
| CLIF-SOFA | **0.0344** | **0.0162** | 0.5988 |  |
| CLIF-C ACLF | **0.0227** | 0.1195 | 0.356 | 0.4908 |
